# Bio-Inspired Blade Serrations: A Review on Owl-Based Strategies for Aeroacoustic Noise Mitigation

**DOI:** 10.3390/biomimetics11050313

**Published:** 2026-05-02

**Authors:** Adalberto Nieto, Nacari Marin-Calvo

**Affiliations:** 1 Department of Mechanical Engineering, Universidad Tecnológica de Panamá, Panama City 0819-07289, Panama; adalberto.nieto1@utp.ac.pa; 2 Facultad de Ingeniería Mecánica, Universidad Tecnológica de Panamá, Panama City 0819-07289, Panama; 3 Centro de Estudios Multidisciplinarios en Ciencias, Ingeniería y Tecnología (CEMCIT-AIP), Panama City 0819-07289, Panama

**Keywords:** bio-inspired, aeroacoustics, wind turbines, serrations, owl wings, trailing edge, leading edge, aerodynamic noise control

## Abstract

The increasing deployment of wind energy has brought renewed attention to aeroacoustic noise generated by wind turbine blades, where broadband noise is primarily associated with vortex shedding at the trailing edge (TE) and leading edge (LE) of airfoils. Owls, particularly *Tyto alba*, exhibit wing morphologies such as serrations, velvet-like surfaces, and fringes that enable silent flight through aerodynamic noise suppression. This study presents a scoping review of the scientific literature on owl-inspired serration strategies applied to aerodynamic airfoils and wind turbine blades. The literature search was conducted across major academic databases, including Scopus, ScienceDirect, SpringerLink, and MDPI, covering publications from 1970 to 2025. A total of 69 experimental and numerical studies focusing on LE and TE serrations was analyzed. The review integrates aeroacoustic analysis with bio-inspired design perspectives. The analyzed studies consistently show that serrated geometries modify vortex dynamics and turbulence structures, leading to measurable acoustic benefits. Experimentally, the largest reductions reported for aerodynamic airfoils reached about 7 dB for both LE and TE serrations, mainly as broadband noise attenuation, in specific frequency ranges. Numerically, the highest reported reduction reached up to 21 dB for a serrated TE configuration, corresponding to spectral SPL reduction mainly below 1.6 kHz. The reviewed studies also indicate that the associated aerodynamic response is strongly configuration-dependent, ranging from limited penalties to measurable changes in lift, drag, power output, or structural loading. Numerical simulations further support experimental findings and highlight the importance of geometric parameters such as serration amplitude, wavelength, and spacing. Overall, bio-inspired serrations represent a promising passive strategy for aeroacoustic noise mitigation in wind turbines, drones, and rotating aerodynamic systems. Future research should focus on the multi-objective optimization of serration geometry, large-scale experimental validation, and the integration of bio-inspired concepts into industrial blade designs.

## 1. Introduction

Wind energy has grown as a central component of renewable energy, but aeroacoustic noise remains a challenge for community acceptance. Among the most notable examples is the silent flight of owls, a phenomenon that has attracted particular interest due to their ability to move without producing the characteristic noise of other birds with similar morphology. This ability is an evolutionary advantage that allows species such as *Tyto alba* to stealthily approach their prey in nocturnal environments [1,2].

In the field of engineering, noise generation continues to be a challenge in rotating systems such as wind turbines, helicopter rotors, fans, and drones. Wind turbine blades generate noise due to the interaction of airflow with the leading edge (LE) and trailing edge (TE). As the electrical power of turbines has increased, so has their sound power, intensifying the acoustic impact on nearby communities [3]. This problem is becoming more relevant considering the global expansion of onshore wind energy, which requires sustainable solutions to reduce noise emissions without compromising efficiency.

The main sources of noise in blades are associated with the aerodynamic load they experience during operation. Among the mechanisms identified, vortex noise is the dominant broadband source, originating from the formation and detachment of turbulent structures from the surface and tips of the blades [4]. Further evidence reported by Gruber et al. [5] indicates that TE noise in airfoils is caused by the interaction between turbulent structures within the boundary layer and the geometric discontinuity at the TE. These insights have motivated the exploration of passive noise control strategies aimed at disrupting such mechanisms.

The noise from wind turbines is not only a technical challenge, but also a social concern. Research has shown that even when sound pressure levels remain within regulatory limits, prolonged exposure can affect the quality of sleep and psychological well-being of nearby communities [6]. This impact has led to the adoption of stricter control criteria, especially during nighttime hours, and the recommendation to establish minimum distances between wind turbines and residential areas.

The operation of a wind turbine generates two main types of noise: mechanical and aerodynamic. The former originates from internal components such as the gearbox, generator, and cooling fans and usually manifests as a tonal sound caused by vibrations or friction. Aerodynamic noise, on the other hand, arises from the interaction of the flow with the blades, resulting from pressure fluctuations, vortex shedding, and turbulence at the LE and TE. Predominantly non-tonal and broadband in nature, this type of noise increases with rotor speed and the intensity of atmospheric turbulence.

Two critical regions can be distinguished in the dynamics of flow around a blade: the leading edge (LE), corresponding to the front edge that receives the incident flow, flow separation (stagnation point: where the flow change direction) that defines the initial pressure distribution over the profile, and the trailing edge (TE), located at the rear, where the flow from both surfaces rejoins and can generate vortex shedding. These areas are responsible for a large part of the aerodynamic noise, which is why they are the focus of current mitigation strategies.

**Figure 1:**
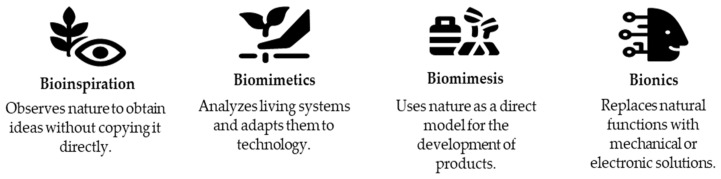
Differences between biomimetics, bio-inspired design, biomimesis and bionics.

Bio-inspiration, understood as the adaptive application of biological principles to engineering solutions, differs from strict biomimetics which seeks to reproduce natural mechanisms with high fidelity and from bionics, which integrates multidisciplinary technological systems inspired by biology. Bio-inspired classic examples include the development of Velcro inspired by plant burs and aerodynamic refinements in high-speed trains modeled after the break of the kingfisher. In our case, bioinspiration emerges as a promising alternative for reducing acoustic emissions without compromising aerodynamic performance. In the aeroacoustics of rotating systems, recent studies have demonstrated that structures inspired by the wings of nocturnal birds, such as the serrations observed in *Tyto alba*, can effectively modulate vortex formation and reduce acoustic emissions without compromising aerodynamic performance. Related strategies have been reviewed in centrifugal fan applications, where bionic adaptations of edges and surfaces lead to significant improvements in noise reduction [7]. Passive strategies, including TE serrations and surface microtextures, constitute the conceptual basis for exploring bio-inspired wind turbine blade designs with enhanced aeroacoustic noise mitigation. In Figure 1, the differences between biomimetics, bio-inspired design and bionics are shown.

Inspired by the morphology of owls, this approach seeks to replicate the natural adaptations that enable their silent flight, in particular the serrations on the LE and the fringes on the TE as passive flow control devices capable of weakening vortex formation and attenuating noise. This paper combines a review of the most relevant advances in this field, highlighting the underlying physical mechanisms, the practical implications of implementing bio-inspired serrations, and the future opportunities for integrating biomimetic design strategies into aerodynamic systems such as wind turbine blades.

## 2. Methodology of the Literature Review

### 2.1. Search Strategy and Study Selection

This study was conducted as a scoping review following the guidelines of the PRISMA Extension for Scoping Reviews (PRISMA-ScR), with the aim of systematically mapping the scientific evidence available on aeroacoustic noise generation, aerodynamic performance, and bio-inspired modifications applied to wind turbine aerodynamic profiles.

The literature search was carried out using major scientific databases and publication platforms, specifically Scopus, ScienceDirect, SpringerLink, MDPI, the digital library of the American Institute of Aeronautics and Astronautics (AIAA), and ABC SENACYT, which was used as an institutional scientific access platform to support the retrieval of relevant publications. The search strategy was developed from combinations of keywords related to aeroacoustics, wind turbine aerodynamics, bio-inspired serrated geometries, and passive noise reduction approaches. The terms employed included expressions such as “aeroacoustic noise”, “aerodynamic noise”, “wind turbine”, “horizontal-axis wind turbine”, “vertical-axis wind turbine”, “wind turbine airfoil”, “wind turbine aerodynamic profile”, “leading-edge serrations”, “trailing-edge serrations”, “bioinspired serrations”, “bio-inspired serrations”, “owl-inspired serrations”, “passive noise mitigation”, “experimental serration tests”, and “noise reduction.” These keywords were combined using the Boolean operators AND and OR; however, the search formulation was not identical across all platforms, since some databases required broader search strings, whereas others allowed more direct and targeted queries depending on their search structure and indexing system. A representative search structure is shown below:

(“aeroacoustic noise” OR “aerodynamic noise”) AND (“wind turbine” OR “horizontal-axis wind turbine” OR “vertical-axis wind turbine” OR “airfoil”) AND (“leading-edge serrations” OR “trailing-edge serrations” OR “bioinspired serrations” OR “passive noise mitigation”).

The search was limited to publications published between 1970 and 2025, in order to cover both the earliest relevant experimental studies on aerodynamic noise and serrations, such as those by Hersh and Hayden (1971) [8] and Soderman (1972, 1973) [9,10], as well as more recent experimental and numerical investigations on bio-inspired surfaces and passive noise control.

All retrieved references were exported and organized in Zotero, where duplicate records were identified and removed prior to screening. As a complementary tool, ResearchRabbit was used to explore citation networks, trace related references, and identify potentially relevant additional studies not captured through the primary database search. This platform also facilitated the visualization of links among related studies and leading authors within the same research line, displaying basic information such as title, year, and abstract, which supported bibliographic traceability and thematic exploration. Records identified through this route were subjected to the same deduplication, screening, and eligibility assessment applied to the rest of the studies.

**Figure 2:**
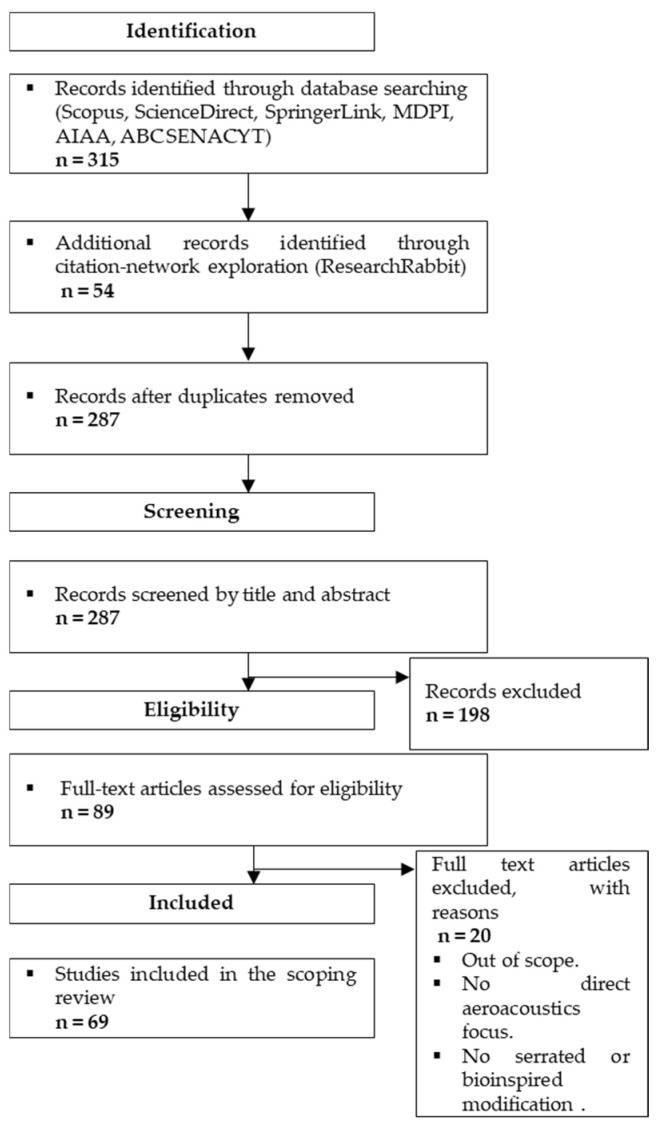
PRISMA-ScR flow diagram of the identification, screening, eligibility, and inclusion process used in this review.

In total, 315 records were identified through database searching and 54 additional records were retrieved through ResearchRabbit. After duplicate removal, 287 unique records remained for title and abstract screening. Subsequently, 198 records were excluded for not fitting the thematic scope of the review. The remaining 89 articles were assessed in full text, of which 20 were excluded for not meeting the predefined eligibility criteria. Ultimately, 69 studies were included in the scoping review. The overall workflow of identification, screening, eligibility assessment, and inclusion is presented in Figure 2, following the structure of a PRISMA-ScR flow diagram.

### 2.2. Eligibility Criteria and Thematic Classification

The inclusion criteria were defined to ensure that the selected literature was directly relevant to aeroacoustic noise mitigation in aerodynamic profiles and wind turbine configurations. Peer-reviewed journal articles and conference papers were included when they addressed at least one of the following aspects: aeroacoustic noise generation in aerodynamic profiles or wind turbine blades; the use of leading-edge (LE) serrations, trailing-edge (TE) serrations, or combined serrated configurations; bio-inspired aerodynamic concepts, particularly those derived from owl wing morphology; and studies developed through experimental, numerical, or hybrid approaches.

Among the numerical studies, works based on computational fluid dynamics (CFD) were considered, as well as investigations supported by computational aeroacoustics (CAA), semi-empirical models, and analytical approaches for aerodynamic noise prediction, including formulations such as TNO-Blake, the Ffowcs Williams–Hawkings (FW-H) acoustic analogy, and other aeroacoustic modeling frameworks used to analyze the effect of serrations on sound generation and propagation. Within CFD-based studies, particular attention was given to those employing approaches such as Detached Eddy Simulation (DES), Large Eddy Simulation (LES), Stress-Blended Eddy Simulation (SBES), and Embedded Large Eddy Simulation (ELES), as these are recurrent in the specialized literature on the aeroacoustics of airfoils and bio-inspired surfaces.

Experimental studies included investigations based on wind tunnel testing, aeroacoustic measurements, aerodynamic characterization of profiles with and without serrated geometries, and performance evaluations associated with passive noise reduction strategies. Studies were excluded if they were not directly related to aeroacoustic noise mitigation in aerodynamic profiles or wind turbines, did not address serrated or bio-inspired modifications, did not provide sufficient methodological information for analysis, or did not correspond to peer-reviewed academic documents.

Once selected, the studies were analyzed and organized into thematic research areas to facilitate literature synthesis and structure the discussion of the paper. The classification included the following main topics: leading-edge serrations, trailing-edge serrations, combined serration configurations, owl wing morphology and silent flight mechanisms, aeroacoustic noise generation mechanisms, passive noise reduction strategies, and numerical aeroacoustic modeling. This thematic organization enabled a systematic mapping of the state of the art and provided the conceptual structure adopted throughout the present review.

### 2.3. Conceptual Structure of the Review

To facilitate the interpretation of the reviewed literature, the discussion is structured progressively from the biological inspiration of silent flight in owls to the physical mechanisms governing aerodynamic noise generation in wind turbine blades. The review then examines the available experimental and numerical evidence regarding serrated airfoil configurations, followed by a design-oriented synthesis of geometric parameters influencing aeroacoustic performance.

Finally, the review concludes with a critical assessment of the current state of research, highlighting existing knowledge gaps and identifying future research directions for the application of bio-inspired aeroacoustic concepts in wind turbine technology.

**Figure 3:**
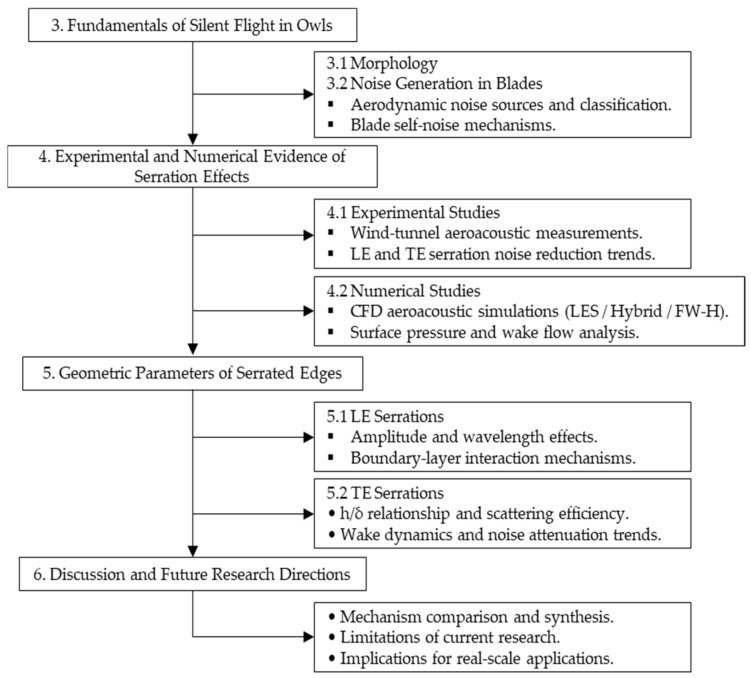
Conceptual structure of the review and organization of the main research topics.

To facilitate the understanding of the conceptual organization of this review, Figure 3 presents the general structure adopted throughout the manuscript. The discussion is progressively developed from the fundamental mechanisms of silent flight observed in owls to the physical processes governing aerodynamic noise generation in wind turbine blades. Subsequently, the available experimental and numerical evidence on the aeroacoustic performance of serrated configurations is examined, followed by a design-oriented synthesis focused on the influence of geometric parameters of leading- and trailing-edge serrations. Finally, the paper concludes with a critical discussion that highlights the main research gaps and outlines future directions for the application of bio-inspired aeroacoustic concepts in wind turbine technology.

## 3. Fundamentals of Silent Flight in Owls

### 3.1. Morphology

The study of morphological adaptations in owl wings as a reference for aerodynamic applications was first introduced by Graham in 1934 [11]. In this work, he conducted a comparative analysis of the feathers and flight behavior of the barn owl (*Tyto alba*) relative to other bird species. Graham observed that distinctive structural features of owl wings are associated with reduced noise generation during flight and suggested their potential as a model for the design of aerodynamic surfaces for aeroacoustic noise control.

Owls have evolved a set of anatomical adaptations that enable near silent flight. Their facial feathers function as acoustic reflectors that channel sound toward the ears, which exhibit an asymmetric arrangement that enhances sound source localization. These morphological features operate in conjunction with specialized neural nuclei in the auditory pathway, thereby improving prey detection capabilities [12]. In addition, the plumage presents microstructures that contribute to the attenuation of aerodynamic noise, particularly at frequencies above 2 kHz, reducing the noise generated during flight within the auditory range of both their prey and the owl itself.

Graham [11] identified three main morphological characteristics associated with the reduction in aerodynamic noise:

1. Comb at the leading edge (LE): Rigid comblike structure located at the front edge of the primary feathers.
2. Fringes at the trailing edge (TE): Flexible structure located along the trailing edge of the wing.
3. Velvet-like dorsal surface: Region covered by fine down that modifies the interaction between the flow and the wing surface.

**Figure 4:**
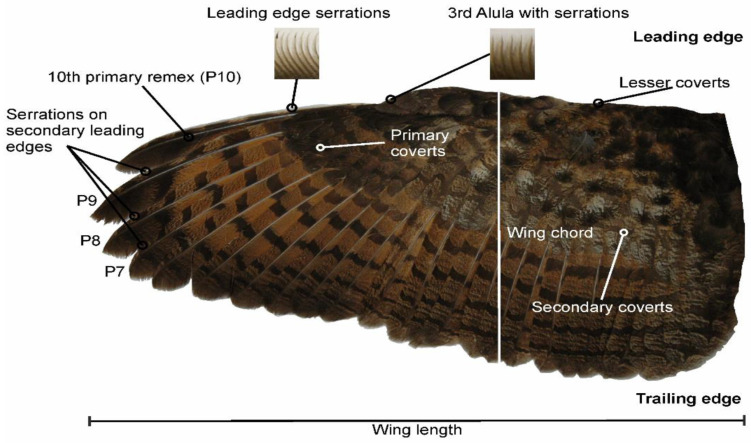
Wing of B. bubo, showing leading-edge serrations on the 10th primary remex and the third alula, as well as on the ninth, eighth, and seventh primary remiges. Ref. [13] Reproduced from Weger and Wagner (2016), licensed under CC BY 4.0.

In Figure 4, the wing of B. bubo is shown, highlighting leading-edge serrations on the 10th primary remex and the third alula, as well as on the ninth, eighth, and seventh primary remiges [13].

These characteristics modify the development of the flow around the wing, altering the formation of vortical structures and reducing the pressure fluctuations associated with noise generation [2,12,14].

In 1998, Lilley [14] confirmed the presence of these three mechanisms and examined them from an aeroacoustic perspective. His analysis showed that these structures alter the interaction between the airflow and the wing surface, thereby weakening noise generation mechanisms, particularly at frequencies above 2000 Hz. Lilley further showed that the reduction in noise is linked to changes in boundary layer development and to altered pressure fluctuation patterns near the TE.

These results were later complemented by morphological studies conducted by Bachmann et al. [12] and Bachmann and Wagner [15], who carried out geometric characterizations of the primary feathers of the barn owl (*Tyto alba*), using three-dimensional (3D) reconstructions. The analysis revealed variations in the geometry, flexibility, and orientation of the natural serrations, indicating that silent flight arises from the combined effect of multiple morphological features that influence boundary layer development and modify the interaction between the airflow and the wing surface.

**Figure 5:**
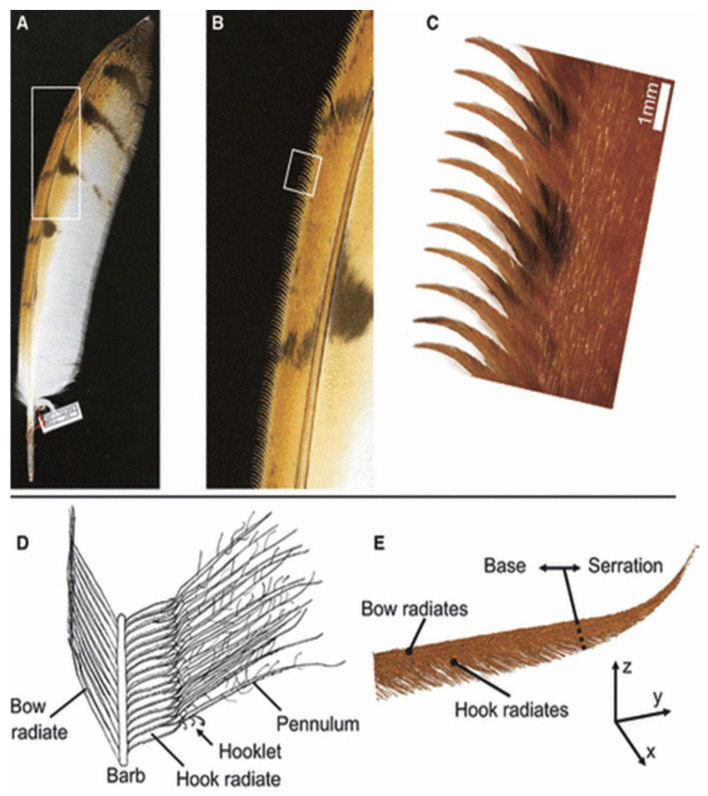
Three-dimensional structure of the serrations on the tenth primary feather of a barn owl wing. (**A**) Photograph of the tenth primary feather. (**B**,**C**) The tips of the outer barbs form the serrations. (**D**) Schematic representation of the anatomy of a barb. (**E**) Three-dimensional reconstruction of a serration using a polygonal mesh. Refs. [13,15] reproduced from Weger and Wagner (2016), licensed under CC BY 4.0.

LE serrations consist of extensions located on the barbs of the primary feathers, particularly on the tenth primary feather (P10-see Figure 5). These structures exhibit curvature, inclination, and torsion, and are oriented opposite to the direction of the incoming airflow. Subsequent studies have demonstrated that these features modify boundary layer behavior and influence aeroacoustic noise generation mechanisms. Bachmann and Wagner [15] and Weger [13] showed that the serrations act as vorticity generators, modifying the structure of the near surface flow. Wei et al. [16] relates owl wing morphology to noise reduction and proposes novel hybrid serration geometries, representing a modern evolution of bioinspired aerodynamic design. Geyer et al. [17] observed noise reductions under the high angle of attack conditions, while Wagner et al. [2] reported that nocturnal species such as *Tyto alba* present more developed serrations, indicating their relationship with noise control during flight.

The velvet-like dorsal surface of owl wings also contributes to aerodynamic noise control. This layer, formed by elongated and flexible pennula located on the dorsal barbs, generates a porous texture that modifies the interaction with the airflow. Geyer et al. [17] demonstrated, through wind tunnel experiments, a reduction in noise at frequencies above 1.6 kHz, attributing this effect to changes in boundary layer development and to a reduction in small-scale fluctuations. Subsequently, Geyer et al. [18] confirmed that this texture contributes to modifying the flow structure even under turbulent conditions. As noted by Lilley [14], this surface does not act as a conventional acoustic absorber. Instead, its function is associated with modifications in the boundary layer structure and with the attenuation of small-scale vortical structures, which constitute a significant source of high-frequency noise. These findings indicate that the velvet-like surface acts as a passive flow control mechanism.

The fringes at the TE are located on the posterior margins of the primary and covert feathers, and consist of unconnected barbs that lack terminal hooks, enabling them to move freely within the flow. Graham [11] established that these structures modify the flow dynamics at the TE, smoothing the transition between the upper and lower streams and reducing the formation of vortical structures associated with noise generation. During hovering or gliding flights, these fringes also contribute to reducing vibrations along the feather edges.

Subsequently, Lilley [14] reinterpreted these fringes as a form of serrated TE capable of reducing acoustic scattering by up to 18 dB, especially when the edge presents a sweep angle of approximately 60°. This estimation is consistent with the theoretical model developed by Howe [19], who demonstrated that serrated edges can reduce aeroacoustics noise radiation as a function of the relationship between the serration height and the acoustic wavelength. Likewise, Bachmann et al. [12] conducted a morphometric study of owl feathers and confirmed the systematic presence of fringes along the inner and outer edges of the primary feathers. These authors described these structures as a morphological specialization that increases the porosity of the wing surface and modifies the interaction between the flow and the TE, contributing to the control of aeroacoustic noise generation.

These morphological characteristics have provided the basis for the development of bio-inspired geometric modifications in aerodynamic profiles, particularly LE and TE serrations, aimed at mitigating aeroacoustic noise in engineering applications such as airfoils and wind turbine blades.

### 3.2. Noise Generation in Blades

In recent decades, the design of wind turbine blades has evolved significantly with the aim of maximizing energy capture, improving aerodynamic efficiency, and increasing electrical output. This progress has led to the development of longer, lighter, and more aerodynamically complex blades. However, a major consequence of these advances has been the increase in noise emissions.

The increase in blade size and rotational speed intensifies the interactions between the airflow and the lifting surface, generating pressure fluctuations and vortical structures that produce broadband and amplitude modulated acoustic emissions. Several studies have reported that wind turbine noise can negatively affect nearby communities, even when sound pressure levels remain within regulatory limits. A study conducted in New Zealand by McBride et al. [6] concludes that wind turbine noise may degrade health-related aspects associated with sleep and psychological wellbeing, recommending conservative nighttime limits and setback distances greater than 2 km to minimize impact.

During operation, different types of noise may be generated [20].

1. Tonal noise: Associated with discrete frequencies, generally caused by mechanical components or non-aerodynamic instabilities.
2. Broadband noise: A continuous distribution of sound pressure above 100 Hz, typically generated by blade turbulence interactions.
3. Low-frequency noise: In the 20–100 Hz range, mainly linked to rotor tower interaction.
4. Impulsive noise: Short acoustic impulses produced by blade interaction with disturbed airflow.

Noise sources can generally be classified into two main categories:

- Mechanical noise: Originates from the dynamic response of components such as the gearbox, generator, and yaw system; it typically exhibits tonal characteristics.
- Aerodynamic noise: Generated by the interaction between airflow and the blades; it is predominantly broadband and tends to increase with rotor speed.

The present work focuses exclusively on aerodynamic noise, as it constitutes the dominant source of acoustic emissions in utility scale turbines under normal operating conditions. Its magnitude depends on blade geometry, orientation, environmental interaction, and operating conditions (wind speed, turbulence intensity, and loading regime), making it a critical area for the development of passive mitigation strategies that do not compromise energy efficiency.

From an aerodynamic perspective, blade noise can be classified into three principal groups [20]: low-frequency noise associated with tower or blade wake interaction; inflow turbulence noise related to atmospheric turbulence fluctuations; and self-noise of the lifting surface, generated directly by airflow along the blade.

**Figure 6:**
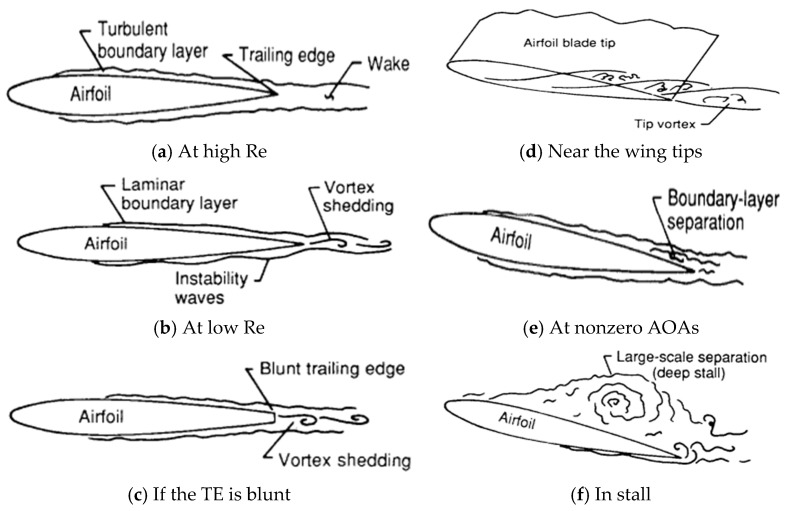
Flow conditions producing airfoil self-noise. Ref. [21] reproduced from Brooks, Pope, and Marcolini (1989), NASA Reference Publication; this work is in the public domain.

To achieve a deeper understanding of the physical mechanisms governing blade self-noise, several authors have proposed interpretations based on boundary layer behavior and flow structure interaction phenomena [21]. Self-noise originates primarily from the interaction between the airfoil surface and the turbulent flow developing around it. The specialized literature identifies multiple physical mechanisms responsible for this phenomenon, summarized in Table 1, while Figure 6 illustrates the general flow conditions associated with each case.

**Figure 7:**
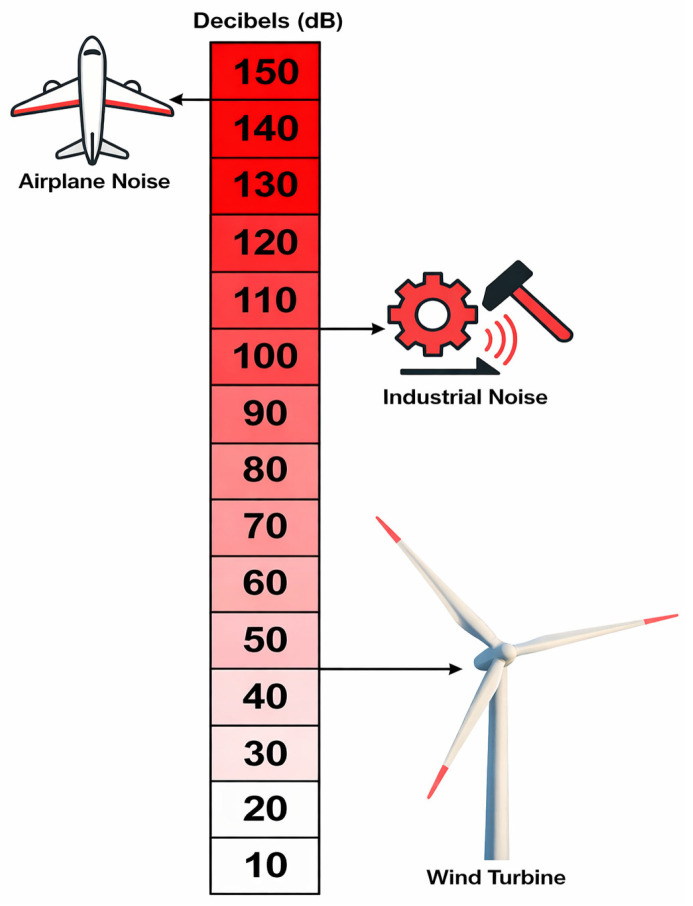
Wind turbines produce noise levels of 35–50 decibels (dB) at 500 m.

TE is typically perceived as broadband noise within the 750–2000 Hz frequency range and arises from the interaction between the turbulent boundary layer and the blade TE. It is widely recognized that the outer 25% of the blade contributes most significantly to both aerodynamic noise and power generation, motivating extensive research on low-noise tip designs. Inflow stall may also produce unstable flow around the lifting surface when the blade operates outside its optimal regime or remains stationary, leading to broadband acoustic emissions. A blunt TE can induce periodic vortex shedding and tonal noise, which can be mitigated by sharpening the edge, although this may introduce manufacturing challenges. Surface imperfections caused by impacts, transport, installation, or lightning strikes may act as acoustic discontinuities and generate additional tonal emissions. Figure 7 illustrates that wind turbines produce noise levels ranging from 35 to 50 decibels (dB) at 500 m.

## 4. Experimental and Numerical Reported Results

Due to the strong dependence of aeroacoustic phenomena on flow conditions and the inherent nonlinearity of fluid behavior, practical and computationally efficient noise prediction methods are essential during the design stage. In this context, aeroacoustic analogies have played a fundamental role, providing the theoretical basis for modern numerical approaches to aerodynamic noise prediction. One of the most significant contributions in this field was made by Lighthill in 1952 [22], who established a fundamental distinction between aerodynamic sound and sound generated by vibrating structures. He demonstrated that sound radiated by turbulent flow can be modeled as a distribution of acoustic quadrupoles, enabling complex fluid dynamic processes to be represented through equivalent sources. Subsequently, in 1954 [23], this formulation was later extended to analyze the acoustic field generated by a turbulent jet. However, this formulation is limited to configurations where solid surfaces can be neglected.

To overcome this limitation, Curle [24] incorporated in 1955 the effect of solid boundaries into the aeroacoustic formulation. From a physical standpoint, solid boundaries influence sound generation by reflecting and deflecting acoustic waves produced by volumetric sources and by restricting these sources to the fluid region outside the solid. Curle introduced a surface integral term representing the acoustic contribution of fluctuating forces exerted by the solid on the fluid, showing that these effects can be modeled as a distribution of acoustic dipoles. This analysis further demonstrated that, at low Mach numbers, dipole sources dominate sound generation.

Later, in 1969, Ffowcs Williams and Hawkings [25] extended the Lighthill–Curle model by considering the acoustic effect of arbitrarily moving surfaces. This extension introduced a third type of acoustic source associated with surface monopoles, thus allowing a more general description of aeroacoustic problems involving moving geometries.

As a result of these developments, aerodynamic sound can generally be represented through the superposition of three types of equivalent acoustic sources: quadrupoles distributed within the flow volume, dipoles associated with unsteady loads on solid surfaces, and monopoles related to the motion of those surfaces. The use of these analogies allows the aerodynamic and acoustic fields to be decoupled, enabling the propagation of computed pressure fluctuations to far-field receivers at a significantly lower computational cost than that required to directly solve the full acoustic field.

**Figure 8:**
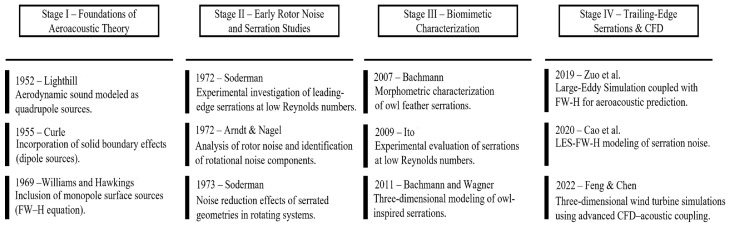
Chronological development of aeroacoustic theory and TE serration research, illustrating the transition from foundational acoustic analogies to modern high-fidelity CFD-based aeroacoustic simulations applied to wind turbine blades [4,9,10,12,15,22,23,24,25,26,27,28,29,30].

The evolution of these theoretical and methodological developments can be understood as a historical progression from foundational aeroacoustic formulations to modern high-fidelity numerical simulations and biomimetic applications. Figure 8 synthetically presents the principal milestones that have shaped this trajectory, providing the conceptual framework for the experimental and numerical studies reviewed in the following sections.

### 4.1. Experimental Studies

The first experimental studies on serrations were reported by Hersh and Hayden [8], who analyzed the acoustic radiation of airfoils and small propellers in laminar and turbulent flow. They demonstrated that properly designed LE serrations suppress tonal noise associated with periodic vortex shedding without significant aerodynamic penalties. Arndt and Nagel [4] later evaluated rotors equipped with serrations, observing reductions in certain components of rotational noise, although accompanied by changes in blade loading. Similarly, Soderman [9], in two-dimensional (2D) airfoil tests, showed that small-scale serrations energized the boundary layer and delayed separation, while rotating configurations [10] exhibited measurable reductions in overall sound pressure level without loss of thrust. Schwind and Allen [30] further reported modifications in laminar separation bubble dynamics and reduced pressure fluctuations at different Reynolds numbers. These findings were consolidated by Hersh et al. [31], who confirmed that serrations reduce the spatial coherence of vortex shedding, attenuating dominant tonal components and decreasing broadband noise.

These results indicate that serrations do not act merely as a superficial geometric modification, but rather as a mechanism capable of altering the spatial organization of the flow near the LE. By fragmenting the regularity of vortex shedding and redistributing energy over a broader spectrum, the most dominant tonal components are attenuated. However, the aerodynamic and acoustic response depends on the configuration, particularly in rotating systems, where interactions with angular velocity and blade loading introduce additional complexities for practical implementation.

Decades later, Dassen [32] evaluated serrated TE in wind tunnel experiments using different airfoil profiles and flat plates, considering variations in angle of attack and flow velocity. Noise reductions between 3 and 8 dB were reported for airfoils and up to 10 dB for flat plates within the 1–6 kHz range, with a strong dependence observed on the alignment of the serrations.

Subsequently, Ito [26] introduced an explicitly bio-inspired approach by replicating the natural serrations of owl wings through toothed elements attached to the LE of airfoils. These experiments showed that such configurations allow lift to be maintained at higher angles of attack under low Reynolds number conditions by delaying flow separation on the suction side. This effect was associated with both aerodynamic improvements and noise mitigation during operation.

In 2011, Gruber et al. [5] experimentally evaluated more than 30 TE sawtooth geometries on an NACA6512 airfoil and found that serrations can reduce far-field sound power by up to 7 dB over a broad frequency range. However, they also observed noise increases of up to about 3 dB at higher frequencies, with the transition between reduction and increase occurring at approximately Stδ = fδ/U0 ≈ 1. Their results also showed that serrations become effective for h/δ > 0.5, reach maximum efficiency for h/δ > 2, and provide greater reductions as λ decreases. Overall, these results indicate that the aeroacoustic benefit of TE serrations depends strongly on their geometric scaling relative to the boundary layer and is not uniformly favorable across the entire spectrum.

Narayanan et al. [33] experimentally investigated sinusoidal LE serrations on flat plates and NACA-65 aerofoils under impinging turbulence. They found negligible noise reduction at low frequencies but significant attenuation in the mid-frequency range (about 500 Hz to 8 kHz), with maximum sound power reductions of about 9 dB for the flat plate and 7 dB for the NACA-65 aerofoil. The study also showed that serration amplitude 2 h was the dominant geometric parameter, while λ had a weaker influence. Similarly, Geyer et al. [34] experimentally evaluated hook-shaped structures installed at the LE of an LS(1)-0413 airfoil, observing noise reductions at low frequencies below 1.6 kHz, along with slight increases at higher frequencies, without appreciable deterioration in aerodynamic performance.

A more detailed analysis of the flow noise interaction was conducted by León et al. [35], who used time-resolved stereo PIV to characterize the boundary layer over flow-aligned TE serrations, revealing modifications in the turbulent structure associated with noise reduction. Wind tunnel tests on the Nordex AD030 (30% relative thickness) and NACA 63-418 (18% thickness) airfoils were combined with field measurements, showing that increasing the flap angle enhanced lift without a significant drag penalty and allowing its impact on power curves and total loads to be quantified [36]. Tlua [37] reported a maximum theoretical reduction of 12.89 dB in overall sound pressure level (OASPL) for an optimum sawtooth TE configuration over the 0.1–10 kHz range, although the corresponding experimental comparisons at 24 m/s and 0° angle of attack showed more modest reductions of about 1–4 dB in the measured far-field spectrum. Likewise, Cao et al. [38] integrated acoustic and aerodynamic measurements with modal analysis through proper orthogonal decomposition (POD), associating reductions of approximately 2 dB with the inhibition of large-scale vortex structures in the suction-side boundary layer.

Ramli et al. (2023) [39] experimentally evaluated an NACA 0012 airfoil modified with LE, TE, and both edge (BE) serrations at Reynolds numbers of 20,000 and 40,000. The term both edge (BE) is used throughout this work to refer to the simultaneous consideration of both the leading edge and trailing edge of the wind turbine blade. Their results showed that the TE configuration reduced drag and improved the lift-to-drag ratio at Re = 40,000, whereas the BL configuration retained the highest lift coefficient. In contrast, the BE configuration produced the poorest aerodynamic response. These results indicate that the aerodynamic effect of serrations is strongly dependent on both configuration and Reynolds number, rather than being universally neutral.

Santamaría et al. (2025) [40] extended the application of bio-inspired serrations to a vertical-axis wind turbine (VAWT) using the DU06-W-200 airfoil. The study considered aerodynamic forces, coefficients, and pitching moments over a wide operational range, observing improvements in the lift-to-drag ratio under near stall and negative angle conditions, as well as an approximate 2% increase in peak performance without compromising aerodynamic stability. This work provides recent evidence of the viability of these configurations in urban applications and under variable operating regimes.

Beyond section-based and wind tunnel investigations, more recent studies have moved toward experimental validation in complete turbines, where the acoustic benefits of TE serrations must be assessed together with their impact on aerodynamic performance and structural loading. In a small-scale horizontal-axis wind turbine (D=3 m), Volkmer et al. [41] applied TE serrations over the outer third of the blade span and validated their effect through extended field measurements of shaft power and overall sound power level (OSWL) under real operating conditions. Their results showed an OSWL reduction of approximately 2 dB, although this acoustic benefit was accompanied by a measurable penalty in shaft power. While the article does not report this penalty explicitly in percentage terms, the corresponding data indicate that the aerodynamic cost was clearly non-negligible, thereby illustrating the practical aeroacoustic trade-off associated with TE serrations. At a larger scale, Zhang et al. (2025) [42] evaluated TE serrations on a 3 MW horizontal-axis wind turbine equipped with 76 m blades under field conditions. Their measurements showed a maximum apparent sound power level reduction of 3.9 dB at 6.5 m/s, while a significant reduction of 2.6 dB was maintained at 8.5 m/s. However, this acoustic improvement was accompanied by a reduction in less than 1% in annual equivalent power generation, corresponding to approximately 25 h, as well as increases of about 2–4% in extreme and fatigue loads. These results highlight that the practical value of TE serrations at full scale must be assessed not only in terms of acoustic benefit, but also in relation to energy yield and structural loading. Table 2 provides an overview of experimental studies on LE and TE serrations.

The experimental investigations reviewed here show that serrated geometries consistently modify boundary layer development and vortex dynamics, leading to measurable reductions in acoustic emissions. However, the associated aerodynamic response is not universally neutral and depends strongly on serration geometry, operating conditions, and turbine configuration. While several studies reported limited penalties or even localized aerodynamic improvements, others revealed reductions in lift, power penalties, or increased loading under specific conditions. These findings indicate that the practical value of serrations cannot be judged solely by the magnitude of noise attenuation, but rather by the balance between acoustic benefit and aerodynamic cost. In this context, the variability observed across experimental configurations highlights the need for predictive tools capable of resolving the coupled flow–acoustic mechanisms in greater detail, thereby motivating the advanced numerical approaches discussed in the following section.

### 4.2. Numerical Studies

The numerical analysis of bio-inspired modifications applied to aerodynamic profiles, both at the LE and TE, has assumed a central role in understanding the mechanisms of aerodynamic noise reduction. Early computational studies were primarily oriented toward evaluating the geometric impact of these configurations on overall aerodynamic performance, employing steady or unsteady RANS models. Although these approaches made it possible to identify general trends in lift, drag, and potential acoustic attenuation, their ability to resolve the turbulent structures responsible for broadband noise was limited. With the advancement of computational resources, a transition toward higher fidelity methodologies occurred, such as LES, DES, and hybrid formulations, enabling a more accurate representation of vortex dynamics, boundary layer edge interactions, and wake development in serrated or microstructured geometries. More recently, the systematic integration of aeroacoustic analogies, particularly the Ffowcs Williams–Hawkings (FW-H) model, has made it possible to obtain detailed spectral predictions and to analyze the frequency dependence of sound attenuation mechanisms. This methodological evolution reflects a progressive shift from predominantly aerodynamic evaluations toward integrated turbulence acoustics frameworks in 2D and 3D configurations, where local geometric effects and spanwise variations play a determining role in the overall aeroacoustic response.

**Figure 9:**
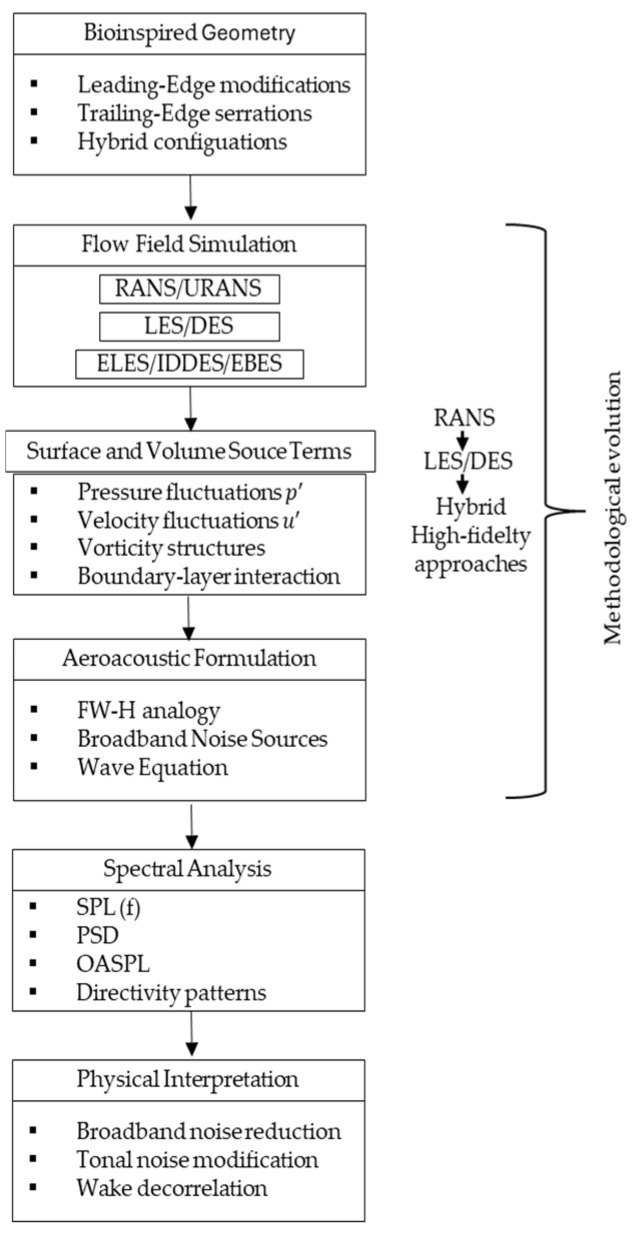
Conceptual framework of numerical aeroacoustic methodologies applied to bio-inspired LE and TE modifications.

With the purpose of contextualizing numerical studies that employ commercial environments widely used in the literature, Figure 9 summarizes the typical methodological workflow adopted in aeroacoustic simulations implemented in ANSYS Fluent 2025 R1, with comparisons to earlier studies conducted using versions released between 2015 and 2024. This scheme is not intended to universally represent all existing computational approaches, but rather to illustrate the most frequent operational sequence when high-fidelity turbulence models are coupled with FW-H analogy within that environment. The representation integrates flow field resolution, source term extraction, and subsequent spectral post-processing, which are stages that constitute the basis of aeroacoustic analysis in numerous recent investigations.

To provide a structured overview of numerical advancements in serrated edge noise research, Table 3 summarizes the main computational studies from 2010 to 2025. The comparison includes edge type, turbulence modeling approach, dimensional framework, acoustic prediction method, and key contributions. This unified presentation clarifies the methodological evolution from low-order and RANS-based models to high-fidelity LES, IDDES, hybrid CFD/CAA, and lattice Boltzmann approaches.

The reviewed studies show that the numerical modeling of serrations does not follow a linear progression toward higher fidelity but instead depends on the physical scale of the problem and the specific objective of each investigation. While isolated airfoil configurations have often relied on LES and hybrid 3D models to resolve unsteady coherent structures associated with broadband noise, other studies have adopted decoupled frameworks based on steady RANS combined with empirical wall-pressure spectrum models and analytical diffraction formulations. These lower cost approaches have proven suitable for extensive parametric studies and geometric sensitivity analyses, particularly for 3D finlet configurations, achieving acceptable agreement with experimental data. In more complex configurations, such as cascades subjected to incoming turbulence, CAA methods solving the linearized Euler equations, coupled with synthetic turbulence generation and the Ffowcs–Williams and Hawkings formulation for acoustic propagation, have been employed. These studies highlight the importance of 3D representations when accurate absolute noise levels are required. ELES and LES simulations at $Re=1.6\times 10^{5}\;$have enabled the association of specific tonal peaks around 2270 Hz with vortex shedding on the pressure side of the TE, showing that longer serrations accelerate the breakdown of these structures and locally modify the flow field in the TE region.

Beyond methodological differences, a clear convergence emerges regarding the dominant physical mechanism of acoustic mitigation. Serrations reduce the spanwise coherence of wall-pressure fluctuations and reorganize the vorticity near the edge, promoting phase interference and the spectral redistribution of acoustic energy. In 3D TE modifications, increasing the serration height decreases the longitudinal scale of turbulent structures and the turbulent kinetic energy in the near wall region, although it may generate additional turbulence in corner regions. Consistently, LES simulations comparing baseline, serrated, comb, and comb-serrated configurations show that the most significant reductions occur at low frequencies particularly below 1.6 kHz with decreases approaching 21 dB in certain configurations. Furthermore, geometric spacing influences the frequency content differently, with more pronounced reductions at high frequencies for smaller spacings and greater effectiveness at low frequencies for larger spacings. In cascade configurations, the spanwise discretization of incoming turbulence has proven critical for obtaining reliable predictions of absolute noise levels, reinforcing the importance of properly representing the 3D coherence of the flow.

Despite the progress achieved, important gaps remain in the literature. Few studies systematically analyze the interaction between serrations and incoming turbulence with different integral length scales in complex 3D configurations. The extrapolation of results from isolated airfoils to full rotor configurations remains limited due to differences in inflow conditions, rotational effects, and variations in aerodynamic load distribution. Although many investigations report acoustic improvements, fewer studies comprehensively quantify the tradeoff between noise reduction and aerodynamic penalties within a consistent comparative framework, particularly when the employed metrics such as spectral peak reduction versus global OASPL and operating conditions differ across studies. In addition, broadband noise behavior has been shown to depend strongly on the angle of attack, affecting both sound level and directivity, with predominant energy concentrations between 90° and 270° azimuth in some cases. This variability in characteristic frequency, tonal amplitude, and radiation pattern indicates that the effectiveness of serrations cannot be assessed solely in terms of overall noise reduction but requires an integrated analysis of local vortex shedding mechanisms, spectral redistribution, and acoustic directivity.

This gap has been partially addressed by Sadeghimalekabadi et al. (2025) [66], who proposed a multi-objective optimization framework for TE serrations by coupling LES, the FW-H acoustic analogy, a feed forward neural network, and a genetic algorithm to optimize simultaneously both the S834 airfoil geometry and the serration shape. Their results showed that the conventional strategy of adding a fixed serration to a reference airfoil is suboptimal, since the joint optimization of airfoil and serration produced a more favorable aeroacoustic balance than optimizing either component separately. At $\alpha =6^{\circ }$, the optimized airfoil with fixed serration increased $\frac{C_{L}}{C_{D}}\;$from 89 to 96 while reducing OASPL from 66 to 64 dB, whereas the simultaneous optimization of airfoil and serration yielded $C_{L}/C_{D}=94$ with a lower OASPL of 62 dB. In addition, the single-objective aerodynamic optimization reached a $\frac{C_{L}}{C_{D}}$ of 109, compared with 89 for the baseline airfoil, illustrating the sensitivity of the design space to the selected optimization target. Overall, the proposed framework provided aerodynamic improvements of about 5–7% together with 1–4% noise reduction relative to the baseline case, while reducing the optimization time by approximately 1/20 through the neural network-based reduced order model. These findings indicate that TE serration performance depends not only on serration geometry itself, but also on its aerodynamic coupling with the host airfoil shape, making integrated optimization a more promising design strategy than sequential add-on approaches.

Beyond studies conducted on isolated airfoil or cascade configurations, some works have begun extending numerical analyses to real blades and complete turbines, incorporating rotational effects, radial load distribution, and atmospheric inflow conditions. However, these investigations remain comparatively limited and do not always establish a systematic connection between the mechanisms identified at airfoil scale and their behavior under real operating conditions.

For full-scale turbines, Van der Velden et al. [67] proposed a multifidelity framework combining BEMT with Lattice–Boltzmann/VLES simulations and the FW-H formulation to predict the noise of turbines with serrated TE. The results show that the reductions observed in 2.5D configurations do not translate linearly to the 3D rotating rotor, leading to smaller far-field reductions. This study also identified that the outer blade regions (r/R ≈ 0.75–0.925) dominate the total acoustic emission and that the flap angle influences pressure fluctuations: moderate values (−5.5°) allow reductions of up to 4 dB around 200 Hz, whereas larger angles can generate local increases near 1 kHz. In addition, pressure fluctuations close to the serrated surface act as the main source of scattered noise. For smaller scale turbines, Sesalim and Naser [68] evaluated an “owl airfoil” applied at the tip of an NREL Phase VI turbine using ANSYS Fluent with the Broadband Noise Sources model, reporting reductions of up to 4 dB at higher inflow velocities, associated with modifications of the velocity field along the outer blade region and in the tip vortex area, although local increases were observed at lower velocities, indicating the need for further geometric optimization. In a related approach, Feng et al. [30] analyzed a bionic blade inspired by owl wings with a non-smooth LE and a curved serrated TE using LES coupled with the FW-H acoustic analogy. Their results showed sound pressure level reductions of up to 6.9 dB and an overall noise reduction of about 8.63%, associated with the conversion of large-scale vortices into smaller structures and faster dissipation of the TE vortex population, with a limited aerodynamic penalty of about 2.13% in the power coefficient. Mozafari et al. [69] extended the analysis to a ducted wind turbine with bio-inspired blade sections derived from a 3D owl wing scan, reporting good aerodynamic agreement with experiments, a 6.4% increase in thrust coefficient, and an approximate 8 dB noise reduction associated with a more uniform pressure distribution and downstream flow modifications, while the radial directivity of the sound remained largely unchanged.

## 5. Geometric Parameters of Serrated Edges

### 5.1. LE Serrations

Before analyzing the aerodynamic effects of LE serrations, it is useful to define the main geometric parameters used to describe these structures. Table 4 shows geometric parameters of serrated edges.

Experimental results indicate that serration size directly influences aerodynamic performance. In general, smaller serrations are more effective at improving lift and delaying flow separation when installed near the LE. In many studies, these dimensions are analyzed in nondimensional form using geometric relations such as h/c, which allows different configurations to be compared independently of the airfoil size.

The spacing between teeth λ also affects the flow behavior. A reduction in the spacing between serration elements has been associated with a decrease in surface pressure fluctuations, which contributes to reducing flow instabilities in low Reynolds number regimes. This parameter also determines the density of serrations along the LE, where smaller values of λ imply a larger number of teeth. Such fine distributions have shown similarities to the microstructures present in owl wings, which exhibit dense serrations along the LE and constitute one of the main inspirations for the design of these bio-inspired geometries.

**Figure 10:**
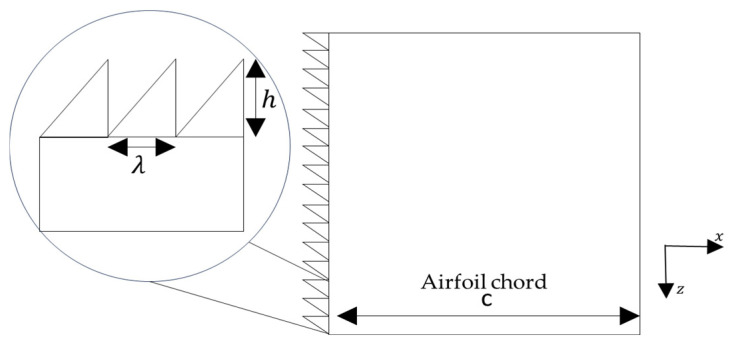
Schematic representation of the geometric parameters used to describe LE serrations, including the serration height h, wavelength λ, and the airfoil chord c.

Another relevant parameter is the position of the serrations along the airfoil chord x/c, as it determines the point where these structures interact with the incoming flow. Serrations located near the LE can modify the flow development in this region and alter the locally generated pressure fluctuations. Likewise, the inclination angle θ and the geometric shape of the serrations (straight, curved, or hook-shaped) also influence the flow structure around the LE and, consequently, the aerodynamic behavior of the airfoil. In several studies, serration geometries are represented using periodic sinusoidal profiles, which provide a simplified mathematical description of the serrated edge.

(1) $$y\left(x\right)=h\;\sin\left(\frac{2\pi x}{\lambda }\right)$$

where the amplitude defines the serration height and the wavelength determines the spacing between consecutive teeth (see Figure 10).

### 5.2. TE Serrations

The design of TE serrations has been investigated as a passive strategy to reduce broadband aerodynamic noise without significantly compromising aerodynamic performance. Among the geometric parameters commonly used to describe these structures are the serration height 2h, the wavelength or spacing between teeth λ, the deflection angle or flap angle β, and the relationship between the serration height and the boundary layer thickness δ.

The serration height 2h determines the interaction between the turbulent boundary layer and the serrated edge. Several experimental studies have shown that noise reduction occurs when the serration height is comparable to or greater than the boundary layer thickness. In many configurations, conditions such as 2h≳δ or even 2h≈4δ have been reported, allowing the turbulent structures to interact directly with the serrations.

Another relevant parameter is the wavelength λ, which together with the height defines the geometric ratio h/λ. This ratio is often used as a design criterion for TE serrations. Values of h/λ greater than 0.5 are typically required to achieve noticeable broadband noise reductions, while larger values tend to promote destructive interference of the acoustic waves generated along the serrated edge.

**Figure 11:**
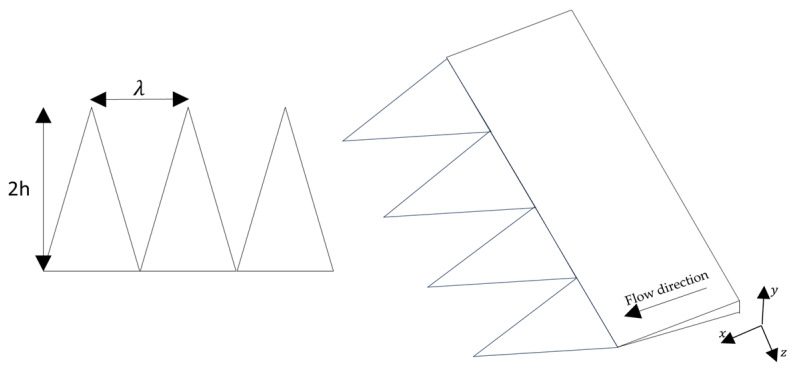
Geometric definition of TE serrations showing the serration height 2h and wavelength λ.

In addition to these parameters, the deflection angle or flap angle β can also influence aerodynamic behavior and noise generation. Moderate deflection angles may increase the lift coefficient while maintaining acceptable drag levels, whereas larger deflections can lead to aerodynamic penalties. For this reason, the combined selection of 2h, λ, and β is an important aspect in the design of TE serrations for noise reduction (see Figure 11).

## 6. Discussion

The reviewed studies show that serrations applied to aerodynamic profiles modify the interaction between the turbulent boundary layer and the airfoil edge, with direct implications for aerodynamic noise generation. TE serrations have been widely investigated as a passive strategy to reduce broadband noise associated with the scattering of turbulent pressure fluctuations at the airfoil edge. From a physical standpoint, noise reduction is related to changes in the acoustic scattering process occurring at the TE. In a straight edge, turbulent boundary layer structures interact relatively coherently with the airfoil edge, promoting stronger acoustic radiation. In contrast, a serrated edge introduces geometric variations that generate phase differences along the edge, reducing the spatial correlation of pressure fluctuations and therefore decreasing the acoustic radiation efficiency of the turbulent flow.

The studies analyzed in this review consistently report noise reductions typically between 3 and 8 dB when TE serrations are used [28,45,46,59,62], reaching values close to 10 dB in optimized configurations [43,48,52]. These reductions depend largely on the geometric proportions of the serration, particularly the tooth height h and spacing λ. When these parameters fall within appropriate ranges, serrations promote the formation of streamwise vortical structures that enhance wake mixing and reduce the coherence of pressure fluctuations along the airfoil edge. However, although serration generally reduces noise at low and mid frequencies, some studies have reported increases in the high-frequency range associated with the generation of smaller turbulent structures.

The importance of the local flow modification near the TE has been highlighted by several numerical investigations. Jones and Sandberg [44] and Avallone et al. [47] both showed that the reduction in TE noise is mainly associated with changes in the local scattering process rather than with significant modifications of the incoming boundary layer. Jones and Sandberg [44], using DNS, reported reductions of approximately 6–10 dB and attributed the effect to the breakup of large turbulent structures and the formation of horseshoe vortices near the serrated edge. In contrast, Avallone et al. [47], using a compressible Lattice–Boltzmann approach, demonstrated that the acoustic benefit can be further enhanced through geometric refinement of the serration, as a curved configuration achieved about 2 dB additional reduction compared with a conventional sawtooth geometry. These results suggest that the 3D flow dynamics near the serration root play an important role in determining the overall level of noise attenuation.

Similar observations emerge from the studies of Zuo et al. [27] and Wang et al. [50]. Zuo et al. [27], using an ELES/FW-H framework, compared a baseline NACA-0018 airfoil with serrated configurations of different lengths and showed that the serrations weaken a tonal peak near 2270 Hz by disrupting the periodic vortex shedding at the TE. However, the overall reduction was relatively modest, with an OASPL decrease of about 1.5 dB. By contrast, Wang et al. [50] reported a much larger reduction of 9.94 dB in a bio-inspired configuration that combined TE serrations with a modified airfoil profile and LE waviness. While this result demonstrates the potential of integrated biomimetic designs, it also indicates that the acoustic benefit cannot be attributed solely to the serrated TE.

Additional insight into the underlying mechanisms has been provided by studies focusing on wake dynamics and numerical modeling strategies. Cao et al. [28] proposed a simplified model that represents serrations through equivalent momentum sources, showing that similar flow structures and acoustic levels can be reproduced without explicitly resolving the serrated geometry. Their results indicated that serrations induce pairs of counter rotating vortices associated with spanwise pressure gradients, enhancing the mixing between the pressure and suction sides and reducing pressure fluctuations in the wake. This approach was later extended by Cao et al. [58] using IDDES coupled with the FW-H acoustic analogy, confirming that the aeroacoustic effect of serrations can be captured even with simplified geometric representations. Complementary results were reported by Chen et al. [61] who performed fully resolved LES/FW-H simulations and observed noise reductions of up to 6 dB at frequencies below 10 kHz. Their analysis showed that serrations reorganize the wake vortex structure, transforming spanwise vortices into larger streamwise structures and reducing the spanwise coherence of pressure fluctuations responsible for acoustic radiation.

In addition to TE modifications, both experimental and numerical evidence indicate that LE serrations can also produce relevant aeroacoustic reductions under specific flow conditions. Wind tunnel experiments have reported typical noise decreases between 3 and 7 dB [9,33,34,42] for airfoil configurations and values approaching 9 dB [33] for flat plates, particularly at moderate Reynolds numbers and within frequency ranges where small-scale turbulent structures dominate acoustic emission. More recent numerical investigations, based on LES, IDDES and hybrid aeroacoustic approaches, have shown additional reductions on the order of 4 to 6 dB in OASPL [46,56,60,62], and even values exceeding 10 dB [65] in geometrically optimized configurations, highlighting the strong influence of parameters such as serration amplitude, wavelength and irregularity. From a physical standpoint, these structures act by modifying the initial interaction between the incoming flow and the aerodynamic surface, promoting boundary layer energization, redistribution of the vortical organization, and spanwise decorrelation of pressure fluctuations, which contribute to attenuating both tonal components associated with periodic vortex shedding and part of the broadband noise. However, the aeroacoustic and aerodynamic response remains highly sensitive to Reynolds number, angle of attack and aerodynamic loading, particularly in rotating applications such as wind turbines, where tradeoffs may arise between noise mitigation, flow stability and overall aerodynamic performance.

Interest in these configurations is closely related to observations of the silent flight of owls. The wings of these birds exhibit specialized microstructures, including serrations at the LE and flexible fringes at the TE, which modify the interaction between airflow and the wing surface. These natural adaptations have inspired bio-inspired aerodynamic designs aimed at reducing aerodynamic noise in engineering applications such as wind turbines.

Overall, the available experimental and numerical evidence indicates that the effectiveness of serrations depends on a combination of geometric and aerodynamic factors, including tooth height, serration spacing, and their relationship with the boundary layer thickness. Although significant progress has been made in understanding the mechanisms responsible for noise reduction, the complete characterization of these effects remains an active area of research, particularly under 3D flow conditions and in full-scale wind turbine applications.

Taken together, the studies reviewed in this work indicate that serrated aerodynamic edges influence aeroacoustic emissions through multiple coupled mechanisms involving boundary layer turbulence, wake dynamics, and acoustic scattering processes. While the overall effectiveness of serrations has been demonstrated in numerous laboratory-scale investigations, the magnitude of noise reduction varies significantly depending on geometric parameters, Reynolds number, and aerodynamic loading conditions. These findings highlight both the potential and the current limitations of bio-inspired serrations as a passive aeroacoustic control strategy and motivate a comparative assessment of their associated aerodynamic, energetic, and structural implications.

To synthesize these broader implications, Table 5 compiles selected studies from both the experimental and numerical literature that reported acoustic benefits together with explicit aerodynamic, energetic, or structural effects. As shown, some configurations achieve meaningful noise reductions with limited or acceptable penalties, whereas others involve measurable reductions in lift, increases in drag, power losses, or higher structural loads. Conversely, certain bio-inspired arrangements can also provide simultaneous acoustic and aerodynamic improvements. This comparison therefore supports a more critical interpretation of serration performance beyond noise attenuation alone.

## 7. Recommendations for Future Work

Despite the progress achieved in the study of bio-inspired serrations, important gaps remain regarding the definition of optimal geometries, the validity of results beyond simplified configurations, and the transfer of these designs to real operating conditions. In this context, future work should focus on four main aspects.

▪ Further research is needed to clarify the relationship between serration geometry and aeroacoustic response. Although variables such as h, λ, h/λ, and their relation to the boundary layer thickness have been shown to be important, there is still no universal criterion for defining optimal configurations under different airfoil shapes and flow conditions. In particular, future studies should systematically examine whether geometric conditions that have shown promising results, such as $\frac{h}{\lambda }>0.5$ in TE serrations and serration heights comparable to or greater than the boundary layer thickness (2h≳δ or 2h≈4δ), remain effective across different airfoils, Reynolds numbers, and angles of attack. A relevant open question is whether nonuniform spacing, variable serration amplitude, or spanwise irregular distributions can outperform conventional uniform configurations in reducing broadband noise without introducing aerodynamic penalties or high-frequency noise increases.
▪ Known configurations should be evaluated under operating conditions that are more representative of real applications. A large portion of the available results has been obtained from isolated airfoils or quasi-2D configurations, whereas their application to wind turbine blades involves additional effects such as rotation, radial load variation, nonuniform incidence, and atmospheric turbulence. Therefore, further studies are needed to determine to what extent the aeroacoustic mechanisms identified at airfoil scale are preserved, weakened, or modified in 3D and full-scale configurations. Addressing this question would help establish the actual transferability of the wind tunnel and idealized numerical results to realistic rotor operation.
▪ Hybrid configurations combining different biomimetic mechanisms on the same aerodynamic surface should be investigated. Most of the reviewed studies analyze LE serrations, TE serrations, or owl-inspired surface modifications separately. However, the interaction between these geometries could provide additional benefits in terms of flow control and noise reduction, particularly through their combined effect on boundary layer development, flow separation, and acoustic scattering. In this sense, a relevant hypothesis for future studies is that properly integrated combinations of LE serrations, TE serrations, and surface treatments may produce more favorable aeroacoustic responses than each mechanism applied individually.
▪ Future research should explore biomimetic geometries that more faithfully reproduce the natural structures that inspire these designs. Owl wings exhibit irregularities, curvatures, and nonuniform distributions that are rarely represented realistically in engineering configurations. Evaluating whether 3D-printed serrations or geometries derived from more realistic owl-mimetic morphologies can provide additional aerodynamic and aeroacoustic benefits over conventional idealized sawtooth designs remains an open research direction. Incorporating these features into future studies could help reveal mechanisms that are not captured by simplified periodic geometries and may support the development of more effective bio-inspired noise reduction strategies.
▪ Future research should also examine the structural strength and life-cycle performance of serrated geometries under realistic operating conditions. It remains necessary to determine whether configurations with favorable aeroacoustic performance can also withstand cyclic loading, vibration, erosion, and environmental exposure without premature degradation. Geometries with slender tips or extended serration lengths may provide acoustic benefits, but they may also be more susceptible to fatigue and structural damage over time. Clarifying this trade-off is essential for the practical implementation of serrated designs in engineering applications.

## 8. Conclusions

The literature analyzed in this review confirms that bio-inspired serrations constitute a promising passive strategy for aeroacoustic control in aerodynamic profiles. Across the reviewed studies, TE modifications have consistently demonstrated the capability to reduce broadband noise, while structures inspired by the LE of owl wings have shown the potential to modify the incoming flow, reduce pressure fluctuations, and in some cases improve aerodynamic performance under low Reynolds number conditions.

The reviewed experimental and numerical investigations indicate that the aeroacoustic response of these configurations strongly depends on their geometric parameters. At the TE, variables such as serration height, wavelength, their relationship to boundary layer thickness, and deflection angle govern the interaction between turbulence structures and the serrated edge. At the LE, parameters including size, tooth spacing, chordwise position, and geometric shape influence the development of the flow and the pressure fluctuations generated near the LE.

From a physical standpoint, the literature suggests that noise reduction in TE serrations is primarily associated with a decrease in the spatial coherence of pressure fluctuations and with modifications of the acoustic scattering process at the airfoil edge. In contrast, the aeroacoustic influence of LE serrations appears to be more closely related to changes in the incoming flow and to the dynamics of vortical structures developing near the surface. In both cases, biomimetic concepts inspired by owl wing morphology provide a valuable framework for the design of aerodynamic configurations capable of combining flow control with acoustic mitigation.

However, the review also reveals important limitations in the current state of research. Although numerous studies report significant noise reductions in controlled laboratory configurations and isolated airfoils, the extrapolation of these results to full-scale wind turbine blades operating under realistic atmospheric conditions remains challenging. Overall, the literature indicates that bio-inspired serrations have strong potential as a passive aeroacoustic solution; nevertheless, their effective implementation in real wind energy systems requires improved integration between geometric design, deeper physical understanding of the flow mechanisms involved, and validation under representative operating conditions.

## Figures and Tables

**Table 1:** Noise generation mechanisms in aerodynamic profiles [21].

Condition	Description
At high Reynolds numbers (Figure 6a)	Turbulent boundary layers develop over most of the aerodynamic profile, and noise is generated when this turbulence passes over the TE.
At low Reynolds numbers (Figure 6b)	Predominantly laminar boundary layers develop over the profile surface, and their instabilities lead to vortex shedding and acoustic radiation as the flow passes beyond the TE.
When the TE is blunt (Figure 6c)	Vortex shedding in the small, separated flow region constitutes another source of noise.
Near the blade tips (Figure 6d)	Vortex formation near the blade tips produces highly turbulent flows that can emit noise.
At angles of attack different from zero (Figure 6e,f)	Flow separation may occur near the TE, generating noise because of turbulent vorticity shedding. At high angles of attack and under stall conditions, large-scale flow separation can produce low-frequency noise emissions, similar to those generated by a bluff body in crossflow.

**Table 2:** Overview of experimental studies on LE and TE serrations.

	Trailing-Edge (TE)	Leading-Edge (LE)
Key Experimental Contributions	Dassen et al. [32]; Gruber et al. [5], Arce León et al. [35]; Llorente et al. [36], Tlua [37]; Cao et al. [38]; Ramli et al. [39] and Santamaría [40]	Hersh and Hayden et al. [31]; Arndt and Nagel [4]; Soderman [9,10]; Schwind and Allen [30]; Ito [26]; Narayanan et al. [33]; Geyer et al. [34] and Ramli et al. [39]
Reported Dominant Effects	Interaction with the turbulent boundary layer modifies trailing-edge scattering and redistributes wall-pressure fluctuations, contributing to broadband noise attenuation. Typical experimental reductions range from 3 to 8 dB for airfoils and up to 10 dB for flat plates, with far-field reductions of up to 7 dB also reported for optimized sawtooth geometries. The aeroacoustic response depends strongly on geometric scaling relative to the boundary layer, with effectiveness reported for h/δ>0.5 and maximum benefit for h/δ>2. Noise increases of up to about 3 dB may appear at high frequencies, indicating that the benefit is not uniform across the full spectrum. Aerodynamic penalties are often limited in properly scaled configurations, although full-scale studies report measurable trade-offs in power output and structural loads.	Suppression of coherent vortex shedding attenuates dominant tonal components without major aerodynamic penalties in properly designed configurations. Modification of laminar separation bubbles and boundary layer energization can delay separation and reduce local pressure fluctuations. Under impinging turbulence, significant attenuation has been reported mainly in the mid-frequency range (about 500 Hz to 8 kHz), with maximum reductions of about 9 dB for flat plates and 7 dB for NACA-65 aerofoils. Hook-shaped or owl-inspired LE geometries may reduce low-frequency noise below about 1.6 kHz, although slight increases can appear at higher frequencies. The overall response remains sensitive to Reynolds number, inflow conditions, and rotating-system effects such as blade loading.

**Table 3:** Comparative overview of numerical methods for serrated edge noise reduction.

Author	Year	Edge Type	Turbulence Model	2D/3D	Acoustic Model	Key Contribution
Arce-León [43]	2010	TE	k-ε (RANS)	2D and 3D	Proudman–Lilley	Serration increases turbulence anisotropy and reduces constructive pressure interference.
Jones et al. [44]	2012	TE	DNS	3D	Direct noise evaluation	Serrations reduce TE noise amplitude by disrupting large coherent turbulent structures.
Lyu et al. [45]	2015	TE	Analytical model	N/A	Extended Amiet theory	Up to 10 dB reduction via destructive interference mechanisms.
Chen et al. [46]	2015	LE	LES	3D	FW-H	Mid–high-frequency noise reduction via spanwise decorrelation and phase interference.
Avallone et al. [47]	2017	TE	Lattice Boltzmann	3D	Direct far-field (LBM)	Curved serrations achieve ~2 dB additional broadband noise reduction.
Bodling et al. [48]	2017	TE	ILES	3D	Direct spectral prediction	High-frequency noise reduction (~1.8 dB) via spanwise coherence reduction.
Gelot and Kim [49]	2017	TE	Wall-resolved LES	3D	Direct feedback analysis	Tonal noise reduction via disruption of acoustic feedback loop.
Wang and Liu [50]	2018	Hybrid	LES (WALE)	3D	FW-H	~10 dB noise reduction with improved L/D via vortex-scale suppression.
Mayer et al. [51]	2018	TE	RANS	3D	Semi-empirical STE model	Improved STE noise prediction accounting for constructive/destructive interference.
Zuo et al. [28]	2018	TE	ELES	3D	FW-H	Tonal noise suppression with longer serrations reducing overall SPL.
Lyu and Ayton [52]	2019	TE/LE	RANS	2D/3D	Semi-analytical	Modeling framework/rapid prediction model
Shi and Lee [53]	2019	TE	RANS	3D	Empirical WPS model	Efficient TE noise prediction using RANS-based wall-pressure spectra.
Bodling et al. [54]	2019	TE	Wall-resolved LES	3D	Direct spectral prediction	Up to ~10 dB reduction via reduced spanwise coherence and source edge separation.
Tang et al. [55]	2019	TE	LES	3D	Lighthill–Curle	TE noise reduction via suppression of spanwise vortices and pressure fluctuations.
Cao et al. [28]	2020	TE	LES	3D	FW-H	Efficient TES modeling via momentum-source approach with accurate noise prediction.
Buszyk et al. [56]	2020	LE	CAA	3D	FW-H	~6 dB broadband reduction under grid turbulence conditions.
Liu et al. [57]	2021	TE	CAA	3D	CAA	Parametric optimization of TE serrations achieving ~2 dB SPL reduction.
Cao et al. [58]	2021	TE	IDDES	3D	FW-H	General TES model using momentum-source approach with accurate aeroacoustic prediction
Salama and Rocha [59]	2021	TE	ELES	3D	FW-H	Slanted root serrations reduce tonal peak via weakened vortex shedding.
Buszyk et al. [60]	2021	LE	Hybrid CFD	3D	CAA (Linearized Euler)	3D CFD/CAA cascade study showing 4–6 dB broadband noise reduction.
Chen et al. [61]	2021	TE	LES	3D	FW-H	LES study showing up to 6 dB OASPL reduction via spanwise vortex reorganization.
Xing et al. [62]	2023	LE	IDDES	3D	FW-H	IDDES–FW-H study showing up to 6.3 dB OASPL reduction in blunt TE noise.
Ibren et al. [63]	2024	TE	LES	3D	FW-H	Low-Re LES study showing up to 21 dB reduction with serrations.
Ribeiro et al. [64]	2024	TE	LBM + VLES	3D	FW-H	Full-scale turbine simulation with <2.5 dB deviation from field data.
Kaya et al. [65]	2025	LE	LES	3D	APE (Hybrid CFD/CAA)	Irregular LE serrations achieved up to 11 dB reduction in St=0.25–0.75 via spanwise variation.

**Table 4:** Geometric parameters.

Symbol	Description
h	Serration height or length
λ	Spacing between teeth (pitch)
θ	Inclination angle
t	Thickness of the serrated structure
$\frac{x}{c}$	Position of the serration along the airfoil chord

**Table 5:** Comparison of acoustic benefits and associated aerodynamic implications of serrated configurations.

Study	Type	Edge/Configuration	Acoustic Result	Acoustic Metric	Aerodynamic Implication	Main Observation
Ramli et al. (2023) [39]	Exp.	LE/TE/BE	Not directly quantified.	_	At Re = 40,000, TE: $C_{L}^{max}$ ↓ 2.6% vs. BL; $C_{D}^{max}$ ↓ 18.75% vs. BL; BE: $C_{L}^{max}$ ↓ 26.9% vs. BL.	Aerodynamic response depends strongly on serration arrangement and Reynolds number.
Volkmer et al. (2021) [41]	Exp.	TE	~2 dB.	OSWL.	Shaft power decreased; suggests $\Delta P_{m}^{*}$ ≈ −10 to −12% for the baseline-profile turbine and ≈−3 to −5% for the optimized-profile turbine.	Acoustic benefit was obtained at the expense of shaft-power performance.
Zhang et al. (2025) [42]	Exp. + Num.	TE	3.9 dB at 6.5 m/s; 2.6 dB at 8.5 m/s; maximum spectral reduction 4.7 dB at 400 Hz.	Apparent sound power level.	Annual equivalent power generation < 1% lower (~25 h); extreme and fatigue loads ↑ ~2–4%.	Full-scale noise reduction remained effective, but with energy and load penalties.
Wang and Liu (2018) [50]	Num.	Bionic profile + LE waves + TE serrations	Average SPL reduced from 45.24 dB (NACA0012) to 32.70 dB.	Average SPL at 12 monitoring points.	L/D increased from 25.65 to 26.79 at AoA = 5°; more favorable $C_{p}$ distribution at AoA = 16°.	Bio-inspired coupled modifications yielded simultaneous acoustic and aerodynamic improvement.
Tang et al. (2019) [55]	Num.	TE	OASPL reduced from 81 dB to 73 dB.	OASPL (146–10,000 Hz).	$C_{D}$ increased from $1.13\times 10^{-2}$ to $1.27\times 10^{-2}$; pressure fluctuations near the TE were reduced.	Noise reduction was accompanied by a slight drag penalty linked to expanded separation near the serration root.

## Data Availability

No new data were created or analyzed in this study.

## Abbreviations

The following abbreviations are used in this manuscript:

TE	Trailing Edge
LE	Leading Edge
BE	Both Edge
dB	Decibels
Hz	Hertz
Re	Reynolds number
CFD	Computational Fluid Dynamics
FW-H	Ffowcs Williams–Hawkings
LES	Large Eddy Simulation
DNS	Direct Numerical Simulation
LBM	Lattice Boltzmann Method
CAA	Computational Aeroacoustics
DES	Detached Eddy Simulation
RANS	Reynold-Averaged Navier–Stokes
SBES	Stress-Blended Eddy Simulation
ELES	Embedded Large Eddy Simulation
IDDES	Improved Delayed Detached Eddy Simulation
OASPL	Overall Sound Pressure Level
